# Supplementary material for: Structural Characterization of a Polygonatum cyrtonema Hua Tuber Polysaccharide and Its Contribution to Moisture Retention and Moisture-Proofing of Porous Carbohydrate Material
Source: Molecules. 2022 Aug 6;27(15):5015. doi: 10.3390/molecules27155015 (PMC9370567; doi:10.3390/molecules27155015)
Supplement: Supplementary file 1 [file molecules-27-05015-s001.zip › molecules-1822956-supplementary.pdf]

Supplementary Data

Structural Characterization of a *Polygonatum cyrtonema* Hua Tuber Polysaccharide and Its Contribution to Moisture Retention and Moisture-Proofing of Porous Carbohydrate Material

Ling Yu [1*], Yipeng Wang [1], Qingjiu Tang [2], Rongrong Zhang [1], Danyu Zhang [1] and Guangyong Zhu [1]

[1] School of Perfume and Aroma Technology, Shanghai Institute of Technology, Shanghai 201418, China

[2] Institute of Edible Fungi, Shanghai Academy of Agricultural Sciences, Shanghai 201403, China

[*]Corresponding author: Ling Yu

Address: Department of Perfume and Aroma Technology, Shanghai Institute of Technology, No. 100 Haiquan Road, Shanghai, 201418, People's Republic of China

Contents:

Table S1. Analysis on the effect of PCP 85-1-1 on the aroma change of tobacco shreds.

Number	Volatile compounds	Control (μg/g)	Glycerol (μg/g)	PCP 85－1－1 (μg/g)
	Nitrogenous			
1	L-Nicotine	2472.44	1551.27	1886.85
2	2,3-Dipyridyl	20.78	16.37	21.14
3	Indole	7.33	9.68	11.09
4	3-(1-methyl-2-pyrrolidinyl)-, (S)-Pyridine	15.63	-	-
5	3-Methylindole	14.49	11.99	10.85
6	Pyrido[2,3-b] pyrazine	20.78	16.37	21.14
7	9H-Pyrido[3,4-b] indole	20.20	9.03	11.87
	total	2571.65	1614.71	1962.931
	Furans			
8	Furfural	14.43	19.21	27.06
9	Furfuryl alcohol	-	7.39	10.93
10	5-Methyl-2-furfural	12.97	14.73	15.71
11	5-Hydroxymethylfurfural	265.82	238.78	251.57
	total	293.21	280.10	305.25
	Alcohols			
12	Farnesol	11.48	14.5	15.61
13	(Z, Z, Z)-9,12,15-Octadecatrien-1-ol	89.6	87.76	80.69
14	trans-Geranylgeraniol	32.44	20.56	21.8
	total	133.51	122.81	118.1
	Phenols			
15	Phenol	39.23	42.17	48.82
16	o-Cresol	21.54	23.71	21.04
17	4-methyl-Phenol	32.58	35.94	40.8
18	Maltol	12.22	11.92	13.04
19	4-ethyl-Phenol,	8.82	17.43	16.45
20	1,2-Benzenediol	125.70	102.71	139.85
21	2-Isopropoxyphenol	17.38	14.41	13.71
22	1,4-Benzenediol	33.20	31	63.70
23	2-Methoxy-4-vin	8.02	10.5	10.89
24	2,6-Dimethoxyphenol	2.44	4.3	-
25	4-Ethylcatechol	19.84	21.77	25.37
	total	320.96	315.86	393.65

	Ketones			
26	2-methyl-2-Cyclopenten-1-one	2.33	4.15	-
27	3-Hexene-2,5-dione	13.04	22.27	26.11
28	3-methyl-2-Cyclopenten-1-one	15.04	16.27	19.34
29	2-hydroxy-3-methyl-2-Cyclopenten-1-one,	26.07	24.4	26.99
30	2,3-Dimethyl-2-cyclopentenyl phenol-1-one	7.17	7.81	5.8
31	2,3-Dihydro-3,5-dihydroxy-6-methyl-4H-pyran-4-one	58.38	67.86	86.21
32	3,5-dihydroxy-2-methyl-4H-Pyran-4-one,	17.25	10	14.04
33	Megastigmatrienone 2	7.17	8.53	9.23
34	4-(3-hydroxy-1-butenyl)-3,5,5-trimethyl-2-Cyclohexene-1-one	19.73	20.34	22.5
35	1-(2,4,5-triethylphenyl)-ethanone	7.78	8.99	9.31
36	2,3,6-trimethyl-1,4-Naphthalenedione	9.10	5.87	6.01
37	Scopoletin	51.45	42.42	49.27
	total	234.50	238.98	274.8
	Acids			
38	2-Furancarboxylic acid	8.25	-	-
39	Benzoic acid	8.79	14.56	17.19
40	Phenylacetic acid	12.37	21.24	12.67
41	Tetradecanoic acid	4.27	4.05	-
42	n-Hexadecanoic acid	201.54	183.59	176.91
43	(Z, Z)-9,12-Octadecadienoic acid	28.12	27.9	21.95
44	Octadecanoic acid	36.68	23.96	20.81
	total	300.02	275.3	249.54
	Esters			
45	Triacetin	286.41	276.46	294.6
46	Diisobutyl phthalate	14.73	5.9	-
47	Methyl palmitate	11.16	10.79	11.07
48	P-propylene benzaldehyde acetal	58.3	24.68	19.36
	total	370.6	317.83	325.03
	Hydrocarbons			
49	Toluene	33.55	68.57	119.09
50	1,3-dimethyl-Benzene	18.26	22.25	33.67
51	β-Pinene	14.23	-	-
52	1-methyl-2-(1-methylethyl)-	8.34	10.71	10.05

	Benzene			
53	Dl-limonene	34.71	43.43	44.62
54	2-methyl- Naphthalene	7.86	12.09	15.63
55	Caryophyllene	73.73	51.61	36.12
56	o-Hydroxybiphenyl	12.52	15.63	10.00
57	1-methyl-2-pentyl-Cyclopropane	16.68	18.33	18.65
58	Neophytadiene	122.17	121.08	133.58
59	Aciphyllene	15.77	16.11	13.44
60	Heptadecane	6.70	9.99	10.13
61	Eicosane	37.86	30.08	36.60
62	Heneicosane	24.73	21.84	24.64
	total	427.12	441.72	506.72
	Total volatile compounds	4651.61	3607.35	4135.55
